# Potential Application of Small Interfering RNA in Gastro-Intestinal Tumors

**DOI:** 10.3390/ph15101295

**Published:** 2022-10-20

**Authors:** Pasquale Losurdo, Nicolò de Manzini, Silvia Palmisano, Mario Grassi, Salvatore Parisi, Flavio Rizzolio, Domenico Tierno, Alice Biasin, Chiara Grassi, Nhung Hai Truong, Gabriele Grassi

**Affiliations:** 1 Surgical Clinic Unit, Department of Medical and Surgical Sciences, Hospital of Cattinara, University of Trieste, Strada di Fiume 447, 34149 Trieste, Italy; 2 Department of Life Sciences, Cattinara University Hospital, Trieste, Strada di Fiume 447, 34149 Trieste, Italy; 3 Department of Engineering and Architecture, Trieste University, Via Valerio 6, 34127 Trieste, Italy; 4 Pathology Unit, Centro di Riferimento Oncologico di Aviano (CRO) IRCCS, Pordenone, 33081 Aviano, Italy; 5 Doctoral School in Molecular Biomedicine, University of Trieste, 34100 Trieste, Italy; 6 Department of Molecular Sciences and Nanosystems, Ca’ Foscari University of Venice, 30123 Venezia, Italy; 7 Degree Course in Medicine, University of Trieste, 34100 Trieste, Italy; 8 Faculty of Biology and Biotechnology, VNUHCM—University of Science, Ho Chi Minh City 700000, Vietnam; 9 Laboratory of Stem Cell Research and Application, VNUHCM—University of Science, Ho Chi Minh City 700000, Vietnam

**Keywords:** gastro-intestinal cancer, liver fibrosis, siRNAs, delivery systems

## Abstract

Despite the progress made in the diagnoses and therapy of gastrointestinal cancers, these diseases are still plagued by a high mortality. Thus, novel therapeutic approaches are urgently required. In this regard, small interfering RNA (siRNA), double-stranded RNA molecules able to specifically target the mRNA of pathological genes, have the potential to be of therapeutic value. To be effective in the human body, siRNAs need to be protected against degradation. Additionally, they need to target the tumor, leaving the normal tissue untouched in an effort to preserve organ function. To accomplish these tasks, siRNAs have been formulated with smart delivery systems such has polymers and lipids. While siRNA protection is not particularly difficult to achieve, their targeting of tumor cells remains problematic. Here, after introducing the general features of gastrointestinal cancers, we describe siRNA characteristics together with representative delivery systems developed for gastrointestinal cancers. Afterward, we present a selection of research papers employing siRNAs against upper- and lower- gastrointestinal cancers. For the liver, we also consider papers using siRNAs to combat liver cirrhosis, a relevant risk factor for liver cancer development. Finally, we present a brief description of clinical trials employing siRNAs for gastrointestinal cancers.

## 1. Gastrointestinal Cancers

In this review, we describe the potential use of small interfering RNAs (siRNAs) for the treatment of gastrointestinal (GI) tumors focusing on gastric cancer, hepatocellular carcinoma, pancreatic cancer and colorectal cancer. Additionally, as liver fibrosis/cirrhosis represents a major risk for the development of hepatocellular carcinoma, we also describe works employing siRNAs against liver fibrosis/cirrhosis.

The adult human gastrointestinal tract has an average length of seven meters. Furthermore, it comprises additional organs including the pancreas, liver, gallbladder and biliary ducts. As a result of substantial cellular mass and the rapid turnover, gastrointestinal cancers are among the most frequent malignancies, responsible for roughly half of all cancer-related deaths.

The distinct tissues of origin give rise to a diverse set of diseases, such as colorectal cancer, pancreatic carcinoma and gastric cancers, each with specific clinical features. In all cases, an altered activation or deactivation of tumor suppressor or proto-oncogenes genes occurs. These common themes can enhance our collective understanding of these malignancies and could perhaps be exploited therapeutically.

Gastric cancer is the fourth most common cancer and the second leading cause of cancer-related mortality in the world. Late diagnosis and classical therapeutic approaches such as surgery, chemotherapy and radiotherapy make this disease a constantly threatening tumor. In Europe, the five-year survival rate is 25% [1,2].

Hepatocellular carcinoma (HCC) is the most frequent primary liver malignancy and one of the most common malignancies worldwide. According to the National Cancer Institute’s SEER database, the average five-year survival rate of HCC patients in the US is 19.6% but can be as low as 2.5% for advanced, metastatic disease [3,4]. When diagnosed at early stages, it is treatable with locoregional approaches, including surgical resection, Radio-Frequency Ablation, Trans-Arterial Chemoembolization, or liver transplantation [5]. However, HCC is usually diagnosed at advanced stages when the tumor is un-resectable, making these treatments ineffective [3,4].

Pancreatic cancer is uniformly fatal unless it can be surgically resected. Pancreatic cancer remains one with the poorest prognosis among all cancers with a five-year survival of 8.1%. The ten-year survival is 3% [6]. Pancreatic cancer represents the fourth cause of death in females (7%) and the sixth in males (5%). The only meaningful chance for a cure is the surgical resection, but only 15% to 20% of patients have potentially resectable disease at presentation. Furthermore, the prognosis is poor even for those who undergo complete (R0) resection [7,8].

To conclude, colorectal cancer (CRC) is the third most common type of non-skin cancer in both men and women. It is the second leading cause of cancer death in the United States after lung cancer. In 2016, an estimated 134,490 people in the United States were diagnosed with colorectal cancer and 49,190 people died from it [9,10]. Incidence rates of colorectal cancer show a positive relationship with an increasing level of economic development [11]. Even so, the five-year survival rate decreases with lower levels of income, with rates reaching 60% in high-income countries in comparison to 30% or less in low-income countries [12].

The common characteristic among the above pathologies is that they have a poor prognosis, and the majority of therapies could often be useless. Thus, novel therapeutic approaches are urgently needed. In this regard, siRNAs hold great promise, mainly due to the ability to specifically target disease genes and due to the flexibility in modulating a large range of targets. However, because of challenges in stability and delivery, it may take time for the clinical practice to become reality. 

## 2. siRNA Structure, Function, and Delivery

Optimal drugs for the treatment of human cancers, including those of the GI tract, still need to be developed. Among molecules of potential interest, there are nucleic acid-based drugs” (NABDs). These potential “drugs” that include ribozymes, DNAzymes, antisense oligonucleotides (ASOs), aptamers, and small interfering RNAs (siRNAs) [13], base their action on the sequence-specific recognition of the biological target. Because of this property, they have gained the attention of biomedical researchers for decades. Whereas some of the NABDs have been developed from the 1980s (DNAzymes, ASOs) and the 1990s (ribozymes), siRNAs were discovered only in 2001. This year [14], it has been demonstrated that siRNA can specifically down-regulate gene expression in mammalian cells. Since then, the potential therapeutic value of siRNA has simply exploded, becoming the most studied among NABD (122758 published papers vs. the second more studied NABD, i.e., DNAzymes with 33,062 papers; source: https://www.ncbi.nlm.nih.gov/myncbi/ (accessed on 3 July 2022).

**Figure 1:**
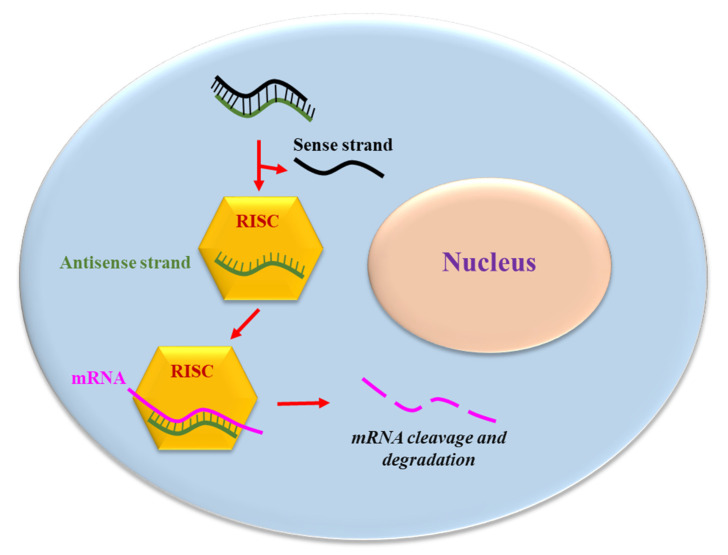
The siRNA mechanism of action. The antisense strand of the siRNA is up-taken by a catalytic protein complex (RNA-induced silencing complex, RISC). The antisense strand drives RISC to a target mRNA, which results in specific, RISC-mediated mRNA degradation.

siRNA consists of two RNA molecules containing 21- and 22-nucleotides; these two RNA strands, sense and anti-sense, are bound together according to Watson and Crick base pairing. In mammalian cells, siRNA is generated by ribonuclease III cleavage from longer double-stranded RNA (dsRNAs) [13]. This cleavage results in the generation of the two strands with 2 nucleotides overhanging at 3′ and 5′ end (Figure 1). The antisense strand of the siRNA guides the RNA-induced silencing complex (RISC) to a complementary cytoplasmic RNA, inducing its degradation; the sense strand is ejected from RISC (Figure 1). However, it cannot be excluded that the sense strand is incorporated into RISC, thus possibly inducing the targeting and destruction of an unwanted RNA. To bypass the risks, the use of single stranded siRNA containing just the antisense strand has been proposed [15]. However, single stranded siRNAs are less potent and stable in biological liquids/cell environments compared to double stranded siRNA.

The therapeutic potential of double stranded siRNA stems from the fact that they can be chemically synthesized and subsequently introduced into the target cells to hit virtually any deleterious RNA. Additionally, siRNAs have a highly sequence specific mode of action: indeed, being partially complementary of the antisense strand to the target RNA prevents RISC action and thus target RNA degradation. This implies that siRNA have an extraordinary discriminatory power with regard to the target RNA. Thus, compared to conventional chemotherapy drugs, they are far more specific and potentially have negligible toxicity, a problem that plagues most chemotherapy drugs. Finally, the generation of synthetic siRNAs is relatively easy, being based on a mathematical algorithm and/or experimental approaches which consider different parameters, including the energy profiles [16], the thermodynamic flexibility of the duplex 3′ end and the melting temperature of the two strands [17].

### 2.1. siRNA Delivery to the Target Cell

**Figure 2:**
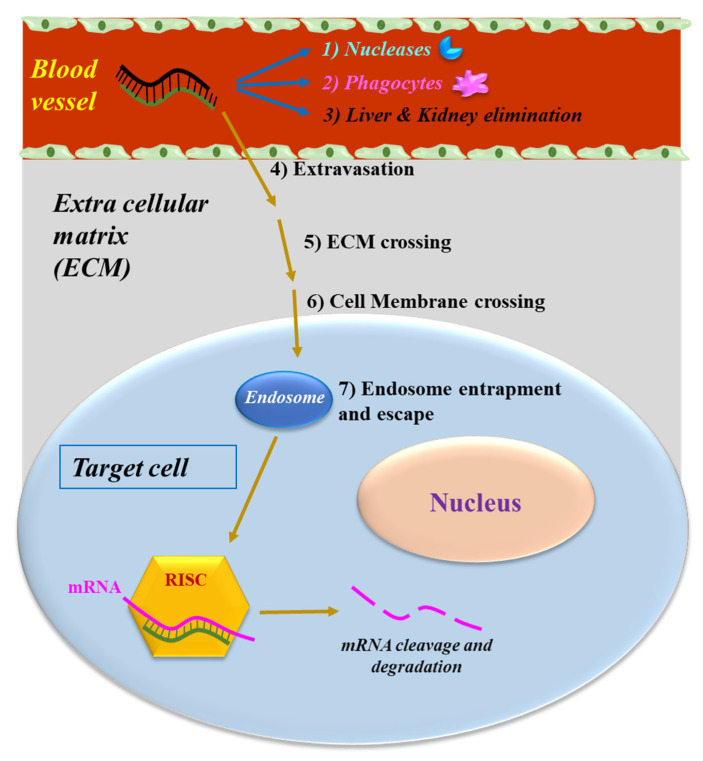
The obstacles to siRNA delivery. Systemically-released siRNAs encounter blood nucleases, which can induce siRNA rapid degradation together with the clearance by phagocytes. Extravasation, cell membrane crossing and endosomal escape are the other barriers to be overcome by siRNAs.

As all NABDs [18,19,20,21,22,23,24,25], siRNAs delivered systemically encounter several different biological barriers. In particular, they can: (1) be degraded by the nucleases present in the blood; (2) be removed by the phagocytic system, (3) be eliminated by the kidneys and/or sequestered in the liver [26] (Figure 2). Moreover, siRNAs have to cross the vessel wall (4), a process called extravasation, and subsequently go through the extracellular matrix (5) to reach the target cell. Here, they need to go through the cellular membrane (6), a process disfavored by the phosphate groups. Indeed, they confer to siRNA a negative electrical charge, which induces the repulsion from the negatively charged surface of the cells. Additionally, the hydrophilic siRNA nature disfavors the crossing of the hydrophobic layer of the cell membrane. Within the target cell, siRNAs need to escape from the endosome [27] (7). These cellular vesicles can trap siRNA, thus preventing the interaction with RISC and subsequently with the target RNA. If not properly addressed by the delivery system, the above obstacles can dramatically impair siRNA effectiveness.

### 2.2. Strategies to Optimize siRNA Delivery

Appropriate delivery vectors can defend siRNA from body elimination and can promote a certain degree of cell targeting. Among the most employed delivery materials, there are lipids and synthetic/natural polymers [13,21,28]. Table 1 summarizes the main features of the discussed delivery materials.

#### 2.2.1. Lipid-Based Delivery Materials

The most well-known type of lipid-based delivery material is the liposome. Liposomes are spherical vesicles constituted by an inner aqueous space surrounded by a bilayer membrane composed of amphiphilic lipids. The aqueous hydrophilic phase can host siRNA, conferring them protection from degradation. Because the outer side of lipids have commonly positive charged moieties, the negatively charged siRNAs can electrostatically bind the outer part of liposomes. Other helpful aspects of liposomes include the fact that they are relatively cheap to produce, can be conjugated with cell targeting moieties and are biodegradable, so they can be easily metabolized and eliminated by the human body. Among the limitations, there is the tendency to accumulate in the liver and lung when administered systemically. However, this feature can be positive in the case of liver/lung targeting. Finally, depending on the specific type of lipids that compose liposomes, a certain attitude to promote inflammation may be present.

A novel class of natural lipid particles termed exosomes became of interest for siRNA delivery. Exosomes, constituted by lipidic nanovesicles 40–100 nm in size, are synthesized by different cells and subsequently released into blood circulation [29]. As they can be isolated from the body fluids of the patient, they show excellent biodistribution and biocompatibility following reinjection into the same patient to deliver therapeutic molecules [30].

#### 2.2.2. Polymer-Based Delivery Materials

Nowadays, many different types of polymers both of natural and synthetic origin have been tested as siRNA delivery materials [20,31,32]. Their use as siRNA delivery materials has grown significantly in recent decades due to the low production/extraction costs. Regardless of the chemical structure of polymers, they contain positively charged chemical groups that allow for electrostatic interaction with the negatively charged phosphate groups present in siRNAs. Among the most used polymers, there is PLGA, a copolymer of polylactic acid (PLA) and polyglycolic acid (PGA), known to be biocompatible/biodegradable and therefore approved by the FDA. Another interesting polymer is Chitosan (CH), obtained via the deacetylation of chitin, present in the exoskeleton of crustaceans. CH is a linear polysaccharide with a carbohydrate backbone containing different amino groups that confers a positive charge, thus enabling the electrostatic interaction with siRNAs. Finally, Hyaluronic acid (HA), is a linear polysaccharide, which can target the siRNA to cancer cells, as often cancer cells express a high level of the HA receptor named cluster determinant 44 (CD44).

The most evident advantage in polymer use depends on the possibility to finely tuning their function(s) via the addition of specific chemical moieties [22]. In this regard, it is possible to improve lysosome escape and improve biocompatibility. For example, polyethyleneimine (PEI) favors the escape of siRNA from an endosome. However, due to a certain degree of cytotoxicity, PEI is often conjugated with poly (ethylene glycol) (PEG), which can minimize cytotoxicity. The possibility to confer to siRNA/polymer complex the ability to reach a defined tissue/cell via the addition of targeting moieties is remarkable. In this respect, small molecules such as folic acid/carbohydrates, antibodies and peptides have been widely employed. The most relevant limitation in polymer use depends on the biodegradability/biocompatibility, bio-distribution, and excretion of the carrier, which often are not optimal. Clearly, depending on the chemical nature of the polymer, these negative aspects can be present and can be attenuated by the addition of smart moieties [28,33,34,35].

#### 2.2.3. Other Delivery Materials

In addition to the above delivery systems, others can be used, such as dendrimers and inorganic nanoparticles. Dendrimers (from the Greek “dendron”: tree, and “meros”: part) are formed by a central core which acts as the root from which a number of highly branched, tree-like arms originate in an ordered and symmetrical fashion [36]. For siRNA delivery, dendrimers are typically constituted by materials with a net cationic surface charge, thus enhabling the electrostatic binding to the negatively charged siRNA. Recently, peptide dendrimers have been used to deliver anti SARS-CoV-2 siRNA [37].

Different inorganic materials have been tested for siRNA delivery. Among these, mesoporous silica are solid materials composed by a honeycomb-like porous structure able to absorb/encapsulate relatively large amounts of siRNA. The high surface area, the large pore volume, the tunable pore size and the good chemical and thermal stability make mesoporous silica very interesting for controlled drug release [38]. Mesoporous silica have been used for siRNA delivery to tumor cells either alone or in combination with conventional chemotherapeutic drugs [39]. Another inorganic material used for siRNA delivery is represented by the gold nanoparticle (GP). GP are amenable for siRNA delivery due to the high surface area to volume ratio, the possibility to undergo multi-functionalization, the facile synthesis, the stable nature and the non-toxic and non-immunogenic nature of gold nanoparticles [40]. For example, a siRNA electrostatically bound to functionalized GP was effectively delivered to prostate cancer cells [41].

In addition to the above mentioned delivery systems, another strategy adopted to promote siRNA delivery and targeting is based on the use of bioconjugation. Bioconjugation consists of the addition to siRNA of biological molecules able to promote cellular uptake, siRNA cell targeting and/or reduce clearance from the circulation. A number of different molecules have ben used in this regard: lipids, peptides, aptamers and sugar moieties [25]. Among lipids, most commonly used is the covalent conjugation with cholesterol used, for example, to target Apolipoprotein B in the liver and [42] myostatin in the murine skeletal muscle [43]. Aptamers are short stretches of single-stranded DNA or RNA that fold into a specific three-dimensional (3D) structure. Their being 3D allows for recognizing a cognate target via shape complementary. They can recognize with high affinity several molecular targets, such as proteins and carbohydrates. Apatamers have been, for example, used to deliver a therapeutic siRNA to prostate cancer cells. The aptamer binds to PSMA (prostate-specific membrane antigen), a cell-surface receptor overexpressed in prostate cancer, while the siRNA targets the expression of survival genes [44]. Peptides are often used in combination with siRNAs due to their ability to confer cell targeting and cell penetration properties. Peptides are typically short and derived from naturally occurring protein translocation motifs. For example, a siRNA has been conjugated to penetratin, short cationic peptides derived from the HIV-1 TAT trans-activator protein to target lung cells [45]. Finally, among sugar residues, N-Acetylgalactosamine (GalNac) has been used for siRNA delivery to the liver. GalNac can target the highly liver-expressed asialoglycoprotein receptor 1 (ASGR1), thus conferring a targeted specificity for the siRNA bound to it. In 2019, an siRNA-GalNac conjugate has been tested in a phase I clinical trial for the treatment of acute hepatic porphyria [46] and approved by the FDA. Moreover, another siRNA-GalNac conjugate has been tested in clinical trials for the treatment of familial hypercholesterolaemia [47] and subsequently approved by the FDA in 2021 (https://www.fda.gov/drugs/news-events-human-drugs/fda-approves-add-therapy-lower-cholesterol-among-certain-high-risk-adults (accessed on 14 October 2022). Finally, the most recent addition to the array of GalNac conjugates in the market is vutrisiran (https://www.fiercepharma.com/marketing/alnylams-next-gen-rna-drug-amvuttra-wins-fda-approval-blockbuster-pfizer-showdown-awaits (accessed on 14 October 2022) used to treat polyneuropathy caused by hereditary amyloidosis in adults.

## 3. siRNAs for the GI Cancers

Targeting drugs to the GI tract can be accomplished via oral, rectal, or endoscopic methods, which can decrease the unnecessary systemic exposure and associated adverse effects of parenteral medications. Despite this, siRNAs delivery to the GI tract encounters the barriers above reported. For this reason, different carriers have been investigated, including biodegradable polymers and lipid-based carriers as below reported.

**Figure 3:**
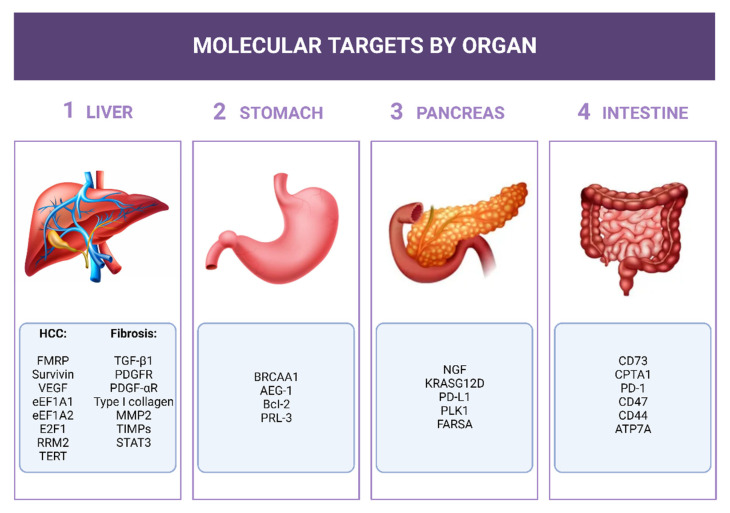
siRNA molecular targets for siRNAs. The mRNAs considered as targets for the siRNA described in the text have been listed close to the respective organ.

Here, we focus on the potential use of siRNAs in the tumor diseases of upper- and lower-GI and of liver fibrosis/cirrhosis due to its role in HCC development. Given the huge number of papers published in the field of GI/siRNAs, here we have selected some of them to give a general overview knowing, however, that other works could also have been mentioned and described. The mRNAs chosen as siRNA targets and described in this review have been summarized in Figure 3.

### 3.1. Potential Role of siRNAs in Upper-GI Cancers

#### 3.1.1. Stomach

The research papers reported in this section have been summarized in Table 2. Cui et al. developed an interesting siRNA delivery approach based on use of the bacteriophage phi29 packaging motor [48]. This is an RNA molecule with a defined structure able to incorporate other molecules such as siRNA (siRNA-pRNA). The structure of pRNA is supposed to protect the siRNA against degradation in a cellular environment. Thus, in this case the authors recurred to the use of a polymer made by RNA for siRNA delivery. To have the possibility to follow the siRNA-pRNA in the cells, a fluorescent die was added (siRNA-pRNA-Fl). In addition, to accomplish targeting towards the gastric cancer cells, a folic acid (FA) molecule was conjugated to siRNA-pRNA-Fl (siRNA-pRNA-Fl-FA). The rationale of FA conjugation is based on the fact that folate receptors (FRs) are over-expressed in cancer cells comparted to in normal cells. As a target for the siRNA, the mRNA of BRCAA1 was chosen (breast cancer-associated antigen 1), which is overexpressed in gastric cancer but far less in normal gastric mucosa cells. In vitro, in the MGC803 gastric cancer cells, siRNA-pRNA-Fl-FA effectively targeted this cell type, but not control cells. Moreover, it reduced MGC803 growth and increased apoptosis. In a subcutaneous mouse model of gastric cancer, it was shown that the intravenous injection of siRNA-pRNA-Fl-FA could effectively localize to the tumor site, reducing cancer cell growth. The data are really promising even if an orthotopic animal model of gastric cancer is deemed necessary to confirm these findings [48].

It has been demonstrated that the proto-oncogene astrocyte elevated gene 1 (AEG-1) is involved in many biological processes, including cell proliferation, survival, apoptosis, invasion and metastasis [52]. AEG-1 regulates tumor cell proliferation through pre-proliferative and anti-apoptotic effects [52]. In gastric cancer tissues, AEG-1 expression was significantly higher than that in normal tissues [49,52], suggesting that it may play an important role in the occurrence and development of gastric cancer. Xu Jian-bo et al. [49] delivered an anti-AEG-1 siRNA by the commercial lipid Lipofectamine 2000 to cultured SGC7901 cancer gastric cells. Compared with non-transfected cells and control siRNA transfected cells, AEG-1 siRNA markedly down-regulated endogenous AEG-1 expression at the mRNA and protein levels, in turn inhibiting cell proliferation. Despite being of potential interest, an in vivo gastric cancer model will be required to fully determine the potential of the described approach.

The B lymphocyte/leukemia-2 (Bcl-2) gene is an oncogene that effectively inhibits cell apoptosis and prolongs cell vitality. Liu et al. delivered the commercial lipid Lipofectamine 2000 an anti BCL-2 siRNA to cultured BGC-823 gastric cancer cells [50]. The authors could observe a decrease in BCL-2 expression paralleled by increased apoptosis when BGC-823 cells were exposed to X-ray irradiation. Despite a more complex animal model being necessary to unravel the therapeutic potential of this approach, this data suggests that BCL-2 silencing may be beneficial following irradiation in vivo.

Cao et al. recently also demonstrated that siRNA-mediated knockdown of the phosphatase of regenerating liver-3 (PRL-3) gene effectively inhibited gastric carcinoma invasion and metastasis [51]. PRL-3 is a metastasis-associated protein for which evidence is accumulating for a role as an oncogene. More in detail, PRL-3 has been associated with cancer progression and metastasis in different human tumors such as pancreatic and ovarian cancers. Moreover, it has been observed that PRL-3 expression is higher in primary gastric carcinoma with peritoneal metastasis compared to peritoneal metastasis-negative gastric carcinoma [51]. siRNAs were delivered by the commercially available commercial lipid Lipofectamine 2000 to the SGC-7901 gastric cancer cells, characterized by a low-grade differentiation. In this cell line, PRL-3 silencing resulted in a significant reduction in cell invasion and metastasis. This effect was most likely due to the downregulation of the matrix metalloproteinases 7 (MMP7), a downstream target of PRL-3. As MMP7 degrades the extracellular matrix, its downregulation is in line with a reduced invasion capacity of SGC-7901. Further in vivo studies will better clarify the effectiveness of this approach.

#### 3.1.2. Pancreas

The work reported in this section has been summarized in Table 3. It has been observed that pancreatic tumors promote neurogenesis via the expression of nerve growth factors (NGFs), which in turn promotes tumor cell survival, proliferation and invasion [53]. Moreover, NGS receptors are actively expressed in pancreatic tumors. Thus, NGF targeting has a potential therapeutic value for the treatment of pancreatic cancer. In this regard, Lei et al. [54] developed a siRNA delivery system based on gold particles (GP), which have several attractive features such as tunable sizes, surface properties and multiple functional capabilities. Being positively charged, GP was easily loaded by the negatively charged anti-NGF siRNA (GP-siNGF). In the cultured pancreatic cancer cell line Panc-1, GP-siNGF, labeled by a fluorescent die, showed excellent uptake, and could effectively escape from an endosome. This last feature allowed for reaching a high concentration in the cytoplasm with the consequent efficient reduction of the mRNA and protein level of the target. In turn, Panc-1 cells displayed significantly reduced proliferation and migration. In vivo, when administered i.v., GP-siNGF showed increased stability of siRNA and prolonged the circulation lifetime compared to naked siRNA. In a mouse subcutaneous model of pancreatic cancer, GP-siNGF efficiently accumulated in the tumor. However, the reason for this observation remains somewhat unclear, as GP-siNGF was not equipped with targeting moieties. Despite this, GP-siNGF could reduce tumor growth compared to control. Interestingly, this observation was confirmed in an orthotopic model and in a patient-derived xenograft model. Finally, GP-siNGF also reduced mesentery metastases.

The K-Ras protein, encoded by the Kirsten rat sarcoma virus (KRAS) gene, transmits the signal from outside the cells to the cytoplasm triggering cell proliferation or differentiation, depending on the specific biological condition. Mutation(s) in KRAS are commonly observed in pancreatic cancer cells, a fact that promotes uncontrolled cell proliferation and metastasis. A common mutation is KRAS^G12D^. Kamerkar et al. [55] developed an exosome-based delivery system for a siRNA directed against KRAS^G12D^ (Exo-si KRAS^G12D^). In vitro, in Panc-1 cells bearing the KRAS^G12D^ mutation, Exo-si KRAS^G12D^ efficiently reduced KRASG12D mRNA; notably, no effect was observed in Panc-1 cells with-wild type KRAS (KRAS^wt^), indicating the specificity of the system. In an orthotopic mouse model of pancreatic cancer, Exo-si KRAS^G12D^ reduced tumor growth compared to controls following i.v. administration. Importantly, animal survival increase was also observed. In line with the in vitro data, Exo-si KRAS^G12D^ had no effects in an orthotopic model of pancreatic cancer with KRAS^wt^. Worth mentioning is the fact that Exo-si KRAS^G12D^ could down-regulate metastasis and prolong animal survival when administered in the advanced state of a pancreatic tumor. This is particularly relevant for potential future application in humans, which often present with advanced forms of the disease.

Programmed death-ligand 1 (PD-L1) is a 40kDa type 1 transmembrane protein that plays a relevant role in down-regulating the adaptive arm of immune systems. Thus, its targeting may be considered a potential strategy to promote antitumor immunity. Based on this concept, Jung et al. [56] targeted PD-L1 via a specific siRNA delivered by the PLGA polymer (PLGA-siPD-L1). By using PLGA-siPD-L1 labeled by a fluorescent die, it was possible to demonstrate the robust uptake in tumor pancreatic cells isolated from a spontaneous orthotopic model of the disease. In parallel, the authors could also show a noticeable down-regulation of PD-L1 mRNA and protein levels. For an in vivo test, a humanized mouse model was used that better recapitulates the human immune system compared to other models. This choice was particularly relevant for having the possibility to study molecules that target a tumor immune microenvironment. The humanized mice were implanted subcutaneously with patient-derived pancreatic tumor cells; the i.v. administration of PLGA-siPD-L1 significantly suppressed tumor growth. Importantly, treated animals had more tumor-infiltrated lymphocytes (TILs), suggesting that tumor growth inhibition was dependent on the increase of TILs whose inhibition by PD-L1 was down-regulated by the PLGA-siPD-L1.

Gemcitabine is used to treat pancreatic cancer. However, often patients develop resistance to Gemcitabine (GEM). Thus, it is relevant to find strategies to reverse the GEM resistance. CXCR4 is a chemokine receptor overexpressed in pancreatic cancer that can promote tumor cell migration. Polo-like kinase 1 (PLK1) represents a family member of serine and threonine kinases highly expressed in pancreatic cancer; moreover, increased PLK1 expression correlates with GEM resistance. Thus, CXCR4 and PLK1 targeting may represent an interesting approach to overcome GEM resistance. Tang et al. [57] developed a strategy based on the use of a polymeric molecule able to antagonize CXCR4 and to carry a siRNA against PLK1 (Pol-siPLK1). In vitro, in the pancreatic cancer cells KPC8060, Pol-siPLK1 achieved nearly 48% target knockdown and 68% in another pancreatic cancer cell line named S2-013. The combination of GEM and Pol-siPLK1showed a significantly improved cell killing effect compared to the single treatment alone, suggesting that Pol was able to antagonize CXCR4 and siPLK1 to down-regulate its target. The author also showed that the effect was synergic in both cell lines and that similar results were obtained in a colony formation assay. In vivo, the authors used two orthotopic mouse models of pancreatic cancer: one syngeneic using KPC8060 and one xenograft using S2-013 cells. In both cases, following intraperitoneal administration, Pol-siPLK1 achieved greatly enhanced accumulation in the tumors. Notably, metastasis was efficiently targeted. Functional studies performed in the orthotopic syngenic KPC8060 revealed an important down-regulation of the growth of both primary tumor and metastasis. Notably, the same group that developed Pol-siPLK1 also prepared a variant of Pol again loaded with an anti PLK1 siRNA which resulted in being effective in reducing the growth of both primary and metastatic tumor pancreatic cells [59].

A particular type of non-coding RNA is represented by CircRNAs, characterized by a covalently closed circular loop structure [60]. It has been proposed that this kind of molecule can act as a sponge for micro-interfering RNAs, thus impairing their regulatory function in gene expression. Emerging data indicate that by altering miRNA regulatory functions, CircRNAs can be implicated in human tumors. Yuan et al. [58] prepared an siRNA able to specifically inhibit the expression of circFARSA, a circRNA derived from exon 5-7 of the FARSA (Phenylalanyl-TRNA Synthetase Subunit Alpha) gene. The siRNA-circFARSA was delivered to target pancreatic cancer cells SW1990, PANC-1 by the commercial lipid Lipofectamine 3000. In SW1990, siRNA-circFARSA effectively reduced circFarsa expression, resulting in increased apoptosis and reduced proliferation. Similar data were reported for the other pancreatic cancer cell lines PANC-1. No effects were, in contrast, observed in both cell lines regarding the cell migratory capacity. In vivo tests were performed with a different delivery material based on porous silicon nanoparticles (pSiNPs). This choice was based on the observation that in vitro pSiNPs gave similar results compared to lipofectamine but with lower toxicity. In vivo pSiNPs-siRNA-circFARSA was injected intraperitoneally in a PDX mouse model of a pancreatic tumor. In addition to confirming the negligible toxicity observed in vitro, pSiNPs-siRNA-circFARSA showed significant down-regulation of tumor growth. These data are particularly relevant for the demonstration of the oncogenic role of circFARSA and open the possibility to consider a novel class of target for therapeutic purposes.

#### 3.1.3. Liver

Chronic infection of hepatitis B/C virus, alcohol abuse and non-alcoholic fatty liver disease are the major causes of liver fibrosis (LF) that invariably lead to the progressive impairment of liver function (cirrhosis) [61]. LF represents a major concern for public health worldwide, with more than 800 million people affected and a mortality rate of approximately 2 million deaths per year [62]. Moreover, liver fibrosis and cirrhosis represent a major risk for the development of hepatocellular carcinoma (HCC). Notably, a number of molecular elements indicate a strict connection between LF and HCC [63]. It is nowadays known that HBV DNA integrates into the hepatocyte genome and that this integration precedes the development of HCC. In this regard, it should be noted that the expression of HBV proteins such as HBx con influence apoptosis in hepatocytes. A similar mechanism occurs for HCV where the core protein NS5A is known to promote HCC development. Additionally, both HBV and HCV affect the expression of micro interfering RNAs (miRNAs) in hepatocytes. Given the role of miRNAs in hepatocytes homeostasis, it is evident that the subversion of their expression can drive the cell towards a cancer phenotype. Not only can the direct effects of HBV/HCV infection promote HCC, indirect effects also can. In particular, the long-lasting hepatic inflammation caused by host immune responses against chronic viral infection can promote liver fibrosis, cirrhosis and HCC progression due to accelerated hepatocyte turnover rates and the accumulation of mutations. Obviously, this is true not only for the viral-induced inflammation but also for all the other pro-inflammatory conditions such as alcohol abuse and non-alcoholic fatty liver disease.

Because of the above reported considerations, LF and HCC represent relevant challenges in the field of gastroenterology for which novel therapeutic approaches are urgently needed. Here we describe some siRNA-based approaches that, in our opinion, are promising for the development of future novel therapeutic approaches.

##### Liver Fibrosis

The work reported in this section has been summarized in Table 4. Irrespective of the etiology, a key element in LF generation is represented by hepatic stellate cells (HSCs) [61,64,65]. In LF, quiescent HSCs trans-differentiate into proliferative and migratory myofibroblasts (cell activation), secreting extracellular matrix (ECM). HSCs, with the features of fibroblasts, are localized in the sub-endothelial space between the basolateral surface of hepatocytes and the anti-luminal side of a fenestrated sinusoidal endothelial cell layer (space of Disse) [64]. In LF, HSCs activation leads to the formation of scar tissue in the space of Disse. Representing the major driver of LF, HSCs targeting is nowadays considered an attractive strategy to down-regulate LF progression and thus liver failure [64]. Notably, deactivating and/or reducing fibrogenic HSCs could be an antifibrotic strategy irrespective of the cause of liver injury [64].

Most of the genes targeted with the purpose to down-regulate LF are implicated in HSC activation and/or are involved in ECM synthesis/degradation. In this regard, transforming growth factor (TGF)-β1 has been considered an interesting target to down-regulate LF. TGF-β1, considered among the most powerful pro-fibrogenic molecules, is heavily implicated in cirrhosis development, being able to regulate ECM gene expression and matrix degradation. Kim et al. studied the effects of TGF-β1 targeting by siRNA in a CCl4-induced murine model of liver fibrosis [66]. The anti TGF-β1 siRNA was cloned in a plasmidic-DNA (p-siRNA), thus ensuring a prolonged expression. The p-siRNA was delivered by liposome through the tail vein. In p-siRNA treated, but not in control animals, TGF-β1 mRNA and protein levels were significantly decreased. This was paralleled by a decrease in the number of cells expressing α-smooth muscle actin (α-SMA) and collagen type I, both known markers of HSC activation [61]. These molecular modifications resulted in the decrease of the histological signs of liver fibrosis and in the reduction of the serum ALT/AST, further indicating an improvement in liver function. Despite these data being interesting, it is questionable whether in humans a plasmid-based delivery for siRNA may be really applied.

The effectiveness of targeting TGF-β1 by siRNA was confirmed in another study by Cheng et al. [67]. In this case, the model considered was the immortalized rat HSC cell line named HSC-T6. Following liposome (the commercial Lipofectamine 2000) mediated delivery of specific anti TGF-β1 siRNAs, the amount of secreted TGF-β1 was significantly reduced compared to control treated cells. The authors also noted that TGF-β1 silencing was paralleled by the down regulation of the tissue inhibitor of metalloproteinases-1 (TIMP-1). This is in line with the knowledge that TGF-β1 inhibits ECM degradation by activating TIMP-1, an inhibitor of MMP, which, in contrast, degrades ECM. Finally, the authors showed that the TGF-β1 silencing resulted in the down-regulation of the expression of α-SMA and collagen type I, both known markers of HSC activation.

Another potential target for LF down-regulation is represented by platelet-derived growth factor (PDGF). The PDGF family is composed of four different components named PDGF-A, PDGF-B, PDGF-C and PDGF-D. A component can aggregate with another one, giving rise to five homo-/heterogeneous dimers (PDGF-AA, AB, BB, CC, and DD). Notably, PDGF-B is considered among the most potent mitogen for HSCs, acting via the interaction with PDGF receptor-β subunit (PDGFR-β). Chen et al. [68] developed an anti PDGFR-β siRNA expressed from a plasmidic DNA under the control of the glial fibrillary acidic protein (GFAP) promoter. The GFAP promoter allowed an HSC restricted expression of the siRNA, thus minimizing the possible side effects of siRNA expression in other liver cell types. In a carbon tetrachloride induced acute injured rat’s liver and in a bile duct ligation (BDL)-induced chronic rat liver injury, the authors could prove the HSC-restricted expression of the siRNA. This was paralleled by a clear relieve of liver injury and hepatic fibrosis in the BDL model. This work is of particular interest because of the HSC restricted expression of the therapeutic siRNA. When combined with a delivery system equipped with smart moieties able to target HSC (via for example the recognition of surface receptors), the safety issue would be excellently addressed.

Lim et al. [69] studied the effects of PDGF receptor-α subunit (PDGFR-α) silencing by siRNA in a co-culture model of hepatocytes (Hep3B) and HSC (LX2). The rationale of this experiment is based on the author’s observation experiment that in the condition of liver fibrosis, hepatocytes increase the expression of PDGFR-α which, via intercellular crosstalk, promotes the overexpression of the same receptor in HSC. Notably, increased expression of PDGFR-α in HSC determines cell activation and proliferation. In vitro Hep3B have an elevated expression of PDGFR-α, a fact that promotes the expression of the same receptor in LX2 and consequently their activation. Co-culturing Hep3B treated by siRNA anti PDGFR-α, delivered by the commercial DharmaFECT, resulted in a significant reduction of LX2 proliferation and expression of α-SMA/collagen type I. This observation indicates the relevance of hepatocytes in the process of liver fibrosis [61] and suggests that the PDGFR-α silencing directly in HSC may potentially be of therapeutic interest.

Following activation, HSCs increase the synthesis of type I collagen, which is produced from the COL1A1 and COL1A2 genes. Calvente et al. [70] generated an anti COL1A1 siRNA and tested its effectiveness in LX2 cells. The authors showed that siRNA-Col1a1, delivered by a lipidic vector, reduced in a dose-dependent fashion of COL1A1 expression and attenuated LX2 migration. siRNA-Col1a1 labeled by a fluorescent die (siRNA-Col1a1-F) was tested in vivo (i.v. injection) in a mouse model of spontaneous biliary fibrosis and in another mouse model where fibrosis was induced by CCl4 treatment. The fluorescent signal was identified in the liver of all mice, a fact not surprising as this kind of lipidic particle tends to be sequestered in the liver [26]. What is, in contrast, interesting is that the accumulation was superior in the fibrotic liver compared to a non-fibrotic control liver. Additionally, the authors observed that siRNA-Col1a1-F mainly accumulated in HSC and Kuepfer cells, both implicated in liver fibrogenesis. Finally, a clear regression of liver fibrosis with no major side effects was reported. These data are certainly of interest especially because they show that it is possible to revert an ongoing fibrotic process, i.e., what is expected to occur in the clinic when most often patients come to the attention of the physician already with variable degrees of fibrosis.

In another work [71], COL1A1 was targeted by a specific siRNA using a sort of “targeted” delivery system. The authors developed a lipid-based delivery system containing vitamin A (Lipid-A). The rationale for this choice is that, compared to the other liver cells, HSC store a high amount of vitamin-A. In cultured human HSC (LI-90), Lipid-A efficiently accumulated into the cells. For the in vivo studies, the CCL4 induced mice model of liver fibrosis was chosen. When administered systemically, Lipid-A effectively accumulated in the a-SMA positive HSCs but not in hepatocytes, proving the targeted ability of the delivery system. When administered combined with the siRNA (Lipid-A-siRNA), the authors could show a robust decrease of COL1A1 mRNA paralleled by a decrease in the protein level. However, no data about the histological regression of liver fibrosis were provided. Finally, it was shown that Lipid-A-siRNA was not toxic, as no significant body weight decrease was observed in treated vs. control mice. Moreover, no major toxicity was detected in the liver by analyzing the circulating levels of transaminases.

##### Hepatocellular Carcinoma

The work reported in this section has been summarized in Table 5. Many strategies have been employed so far to deliver siRNA to HCC cells [20,21]. Here, we focus on those based on systems that can provide a certain degree of targeting, as, in our opinion, these kinds of delivery strategies are the most promising for HCC.

Fragile X mental retardation protein (FMRP) is an RNA-binding protein relevant for the biology of postsynaptic neurons. Despite its clear involvement in the nervous system, evidence is emerging about an involvement in HCC metastasis as well. Therefore, FMRP has been considered an interesting target to combat HCC. Zhao et al. [75] developed an interesting delivery system for siRNA based on the use of carbon dots (CDs). CDs are made of carbon nanomaterial with a size in the range of 1–10 nm. Because of the good biocompatibility and easy synthesis/modification, they have been considered for drug delivery. However, CDs do not have the capability of specifically targeting tumor cells, including HCC. Therefore, Zhao et al., combined CDs with a DNA single-stranded molecule named AS1411, composed of 26 oligonucleotides rich in guanine. Due to its folding, AS1411 can specifically bind to nucleolin (NCL), a protein that takes part in many cellular processes, including DNA replication. Moreover, it is highly expressed in various cancer cells, including HCC. The delivery system (CD-AS1411), loaded with anti FMRP siRNA (CD-AS1411-siFMRP), resulted in being specifically uptaken by the HCC cell model HepG2. Moreover, it effectively down-regulated the expression of FMRP, eventually resulting in the inhibition of migration, invasion and colony formation of HepG2 cells. These promising data need to be further deepened in a more complex in vivo model of HCC.

Asialoglycoprotein receptor (ASGP-R) recognizes and binds galactose and galactosamines residues. While normal hepatocytes express it at low levels, in HCC cells it is over-expressed [83]. Thus, it has been considered an attractive molecule for HCC-specific delivery. For example, ASGP-R has been targeted by a delivery system containing galactose-modified trymethil chitosan-cystein (GTC) [76]. The delivery system was loaded by Survivin and Vascular Endothelial Growth Factor (VEGF) siRNAs (GTC-Surv-VEGF-siRNA). Survivin, overexpressed in most human cancers compared to normal tissues, inhibits apoptosis, thus favoring cancer cell growth; *VEGF* promotes Survivin expression and promotes HCC neovascularization. Following oral administration in a xenograft mouse model of HCC, GTC-Surv-VEGF-siRNA increased tumor apoptosis with no significant un-specific toxicity.

Recently, we [77] developed an siRNA delivery system based on a synthetic polymer containing α,β-poly-(N-2-hydroxyethyl)-D,L-aspartamide-(PHEA) derivatized with diethylene triamine (DETA) and bearing in the side chain galactose (GAL) linked via PEG to obtain PHEA-DETA-PEG-GAL (PDPG). The HCC targeting was demanded by the GAL residue that can interact with ASGP-R. Uptake studies in vitro performed employing HuH7 cells, a human cellular model of HCC, revealing an excellent delivery of siRNA to the cells. Notably, the GAL-free copolymer (PDP) or the chemical block of ASGPR, dramatically lessened the targeting effectiveness. This observation was subsequently confirmed in vivo in a mouse dorsal skinfold window chamber assay. PDPG was then loaded by siRNAs against eukaryotic elongation factor 1A1 (eEF1A1), eukaryotic elongation factor 1A2 (eEF1A2) and the transcription factor E2F1, all involved in HCC [84,85]. In vitro, PDPG-siRNAs significantly decreased HuH7 vitality/number and down-regulated the expression of the target genes. Notably, in immortalized human hepatocytes, a model of normal hepatocytes with reduced ASGPR expression, PDPG barely reduced cell vitality. Finally, in a subcutaneous xenograft mouse model of HCC, PDPG-siRNAs effectively reduced HCC tumor growth compared to controls without significant toxic effects.

In a recent work [78] GalNac, able to target ASGPR, has been used to deliver an anti Pin1 (peptidyl-prolyl cis/trans isomerase 1) siRNA to HCC cells. Pin1, highly expressed in HCC [86], promotes the development of this type of tumor [87]. The authors embedded the GalNac-siRNA complex into a gel that was then injected subcutaneously on the backs of mice. This approach resulted in a siRNA delivery up to 21 days after gel injection. Moreover, the same delivery approach in an orthotopic HCC model, resulted in a significant inhibition of tumor growth.

Epidermal growth factor receptor (EGFR) is a transmembrane receptor tyrosine kinase that, when activated by specific ligands, promotes cell proliferation and survival. Notably, it has a relevant role in HCC [88]. Gao et al. [79] employed liposome-polycation-DNA complexes (TLPD) linked with anti-EGFR Fab’ to deliver the anti-cancer drug Adriamycin (ADR) and an anti ribonucleotide reductase M2 (RRM2) siRNA (ADR-RRM2-TLPD). RRM2 belongs to the ribonucleotide reductase enzyme necessary for DNA replication and cell proliferation in different tumors, including HCC [89]. In an orthotopic model of HCC, ADR-RRM2-TLPD reduced RRM2 expression and significantly inhibited HCC growth.

The transferrin receptor (TfR) is a transmembrane glycoprotein necessary for the uptake of transferrin, the iron-carrying protein circulating in the blood. The expression of TfR is augmented in many cancer cells such as HCC cells [90]. The human insulin receptor (HIR) is a transmembrane glycoprotein highly expressed in cells that are most receptive to the hormone insulin, such as in liver cells. PEGylated liposomes conjugated with antibodies against TfR and HIR (PILPs) [80] were loaded with siRNAs directed against the mRNA of telomerase reverse transcriptase (*TERT*) and *EGFR*, both involved in tumor growth [88,91]. siRNA loaded PILPs resulted in a significant down-regulation of *EGFR* and *TERT* expression paralleled by a reduction of tumor growth in a xenograft mouse model of HCC.

An articulated siRNA delivery system for HCC was developed by Han et al. [81]. The authors used a polymer containing urocanic acid-modified galactosylated trimethyl chitosan (UA-GT) loaded by siRNA against VEGF (siVEGF). The galactose residue allowed the specific uptake in the cell line QGY-7703 via the interaction with ASGP-R. Additionally, urocanic acid residue favored the release from endosome by improving the buffering capacity. When tested in a mouse xenograft subcutaneous model of HCC (i.v administration), the UA-GT-siVEGF effectively diminished tumor growth, as well as tumor blood, thus proving its anti-angiogenic power. This last property is particularly welcome, as HCC is a highly vascularized tumor.

Integrins constitute a group of transmembrane receptors regulating the adhesion of cells to ECM. Following binding to the ligand, they trigger different signal transduction pathways that control several cellular processes, including cell proliferation. The expression of the integrin subtype αvβ3/αvβ5 is increased in the angiogenic endothelium in many malignant tumors [92], including HCC. The binding motif of αvβ3/αvβ5 is represented by the tripeptide arginine glycine aspartic acid (RGD). Wu et al. [82] prepared a complex delivery system based on the use of the polymers PEG and PEI conjugated with RGD (RGD-PEG-*g*-PEI) and loaded with an anti-Survivin siRNA (RGD-PEG-*g*-PEI-siSurv). Following tail vein injection of RGD-PEG-*g*-PEI-siSurv in a subcutaneous mouse model of HCC, the authors observed a clear inhibition of tumor growth with increased tumor HCC cell apoptosis/necrosis. The fact that no significant down-regulation of tumor angiogenesis was observed promotes the speculation that mechanisms different from those predicted could have contributed to the effects of RGD-PEG-*g*-PEI-siSurv.

### 3.2. Roles of siRNAs Target Therapy in Lower-GI Cancers

#### Colorectal Cancer

The scientific works reported in this section have been summarized in Table 6. CD73 is an enzyme able to convert AMP to adenosine; recent evidences indicate that it plays a relevant role in tumor cell growth, migration, angiogenesis and drug resistance [93]. Moreover, it is overexpressed in different types of cancer. Khesth et al. [94] used an siRNA delivery system based on a CH backbone decorated with the peptide TAT and the HA. TAT, derived from the virus HIV, is a cell-penetrating peptide able to favor cell uptake; HA allows the targeting to CD44, overexpressed in many cancer cells. The CH-TAT-HA was loaded with an anti CD73 siRNA (CH-TAT-HA-siCD73) and tested in the colorectal cancer cell line CT26. Confocal microscopic analysis revealed that the TAT-HA dramatically increased particle uptake, resulting in a remarkable reduction in CD73 expression. This, in turn, was paralleled by a significant suppression of cancer cell survival, proliferation and migration. Notably, apoptosis was particularly enhanced. The author also showed that CH-TAT-HA loaded by the anticancer drug doxycycline (DOX) reduced cell vitality. What is important is that the simultaneous administration of CH-TAT-HA-siCD73 and CH-TAT-HA-DOX resulted in a potent inhibition of cell growth, definitively superior to that achieved by the single treatments. This in principle opens the possibility to a combined therapeutic approach. These data were substantially confirmed in vivo in a subcutaneous xenograft mouse model of colorectal cancer. Of note is the observation that the combination of CH-TAT-HA-siCD73 and CH-TAT-HA-DOX also down-regulated tumor neo-angiogenesis. This promising data now requires confirmation in a more realistic orthotopic model of colorectal cancer.

Fatty acid oxidation (FAO) plays a relevant role in cancer, as it can efficiently produce ATP employed in many cellular processes, including cell growth and survival [100]. Carnitine palmitoyltransferase 1A (CPT1A) is a key enzyme of FAO. As it is upregulated in various cancers, it is considered an interesting molecule to target. In this regard, Lin et al. [95] prepared a delivery system based on the use of exosome (EXO) inked to the iRGD peptide (iRGD-EXO). iRGD favors particle extravasation and allows the interaction with integrin αvβ3 (αvβ3), αvβ5 and neuropilin-1 (NRP-1) present on tumor vascular and cancer cells. Notably, the authors observed increased expression of αvβ3 and NRP-1 in cancer tissues compared with noncancerous tissues, thus indicating the cancer targeting potential for iRGD-EXO. iRGD-EXO, loaded with an siRNA anti CPT1A (iRGD-EXO-siCPT1A), showed increased uptake in sw480-lohp and HCT116 compared to EXO-siCPT1A, which lacks the targeting moiety iRGD. This observation was confirmed in vivo in a xenograft subcutaneous mice model of colorectal cancer where iRGD-EXO-siCPT1A accumulated in the tumor starting from 6 h following i.v. administration. In contrast, only minimal accumulation was observed for EXO-siCPT1A. Both in vitro and in vivo, iRGD-EXO-siCPT1A reduced COT1A expression, thus down-regulating fatty acid oxidation and ATP production. In turn, this reduced cell growth and promoted apoptosis. Finally, the authors showed that iRGD-EXO-siCPT1A could reverse resistance to the drug oxaliplatin, an observation in line with the association between CPT1A upregulation and oxaliplatin resistance. Together, these data support the rationale to target CPT1A as a strategy to down-regulate colorectal cancer growth.

PD-1 is an immunosuppressive receptor able to down-regulate T cell activation via the binding to its ligand Programmed death receptor ligand (PD-L1). While PD-1 is mostly expressed on the surface of immune cells, PD-L1 is predominately expressed by tumor cells [101]. Thus, the interaction PD-1/PD-L1 is responsible for the occurrence of the immune escape of tumor cells. Recently, Lu et al. [96] explored the targeting of PD-1 by a specific siRNA delivered via attenuated Salmonella. This is a rather original delivery system where a plasmid, coding for the sequence of the siRNA of interest, is employed to transform attenuated Salmonella, which is used as an expression carrier for the siRNA. In vivo, in a mice xenograft subcutaneous model of colorectal cancer, the Salmonella/siPD-1 efficiently down-regulated target gene expression and, in turn, tumor growth. Additionally, the authors observed increased apoptosis of colorectal cancer cells and migration impairment. Finally, it was observed that Salmonella/siPD-1 could potentiate the antitumor effects of the drug chloroquine, thus suggesting the possibility of a future combined therapeutic strategy. It remains to be understood how applicable to human beings the employment of a delivery system based on attenuated Salmonella is.

The upregulation of CD47 by tumor cells represents a defense mechanism against clearance by the immune system as it provides a “self” signal which allows the escape from immune cells elimination [102]. The optimal removal of cancer cells is, however, necessary to upregulate the expression of the prophagocytic signal calreticulin (CRT) on tumor cells [103]. Based on these concepts, Zhang et al. [97] developed a PLGA-based delivery system for an anti CD47 siRNA (siCD47), combined with the drug mitoxantrone (MTO) that can induce CRT exposure on the surface of tumor cells. After proving in vitro and in vivo in melanoma tumor models that PLGA-siCD47-MTO can be efficiently up taken by tumor cells with the consequent reduction of tumor growth, the authors investigated a xenograft mouse model of colon cancer employing the colon cancer cells CT26. It was shown that the monotherapies with either PLGA-siCD47 or PLGA-MTO achieved approximately 51.4% and 34.9% inhibition rates versus PBS treatment. Interestingly, the administration of siCD47 and MTO in the same delivery particles (PLGA-siCD47-MTO) achieved 85.2% inhibition, suggesting clearly improved anticancer activity likely due to the simultaneous down-regulation of CD47 and upregulation of CRT. In parallel, it was shown that PLGA-siCD47-MTO markedly increased intratumor infiltration of macrophages and T lymphocytes, both involved in cancer cells clearance. This further proves the expected mechanism of action of PLGA-siCD47-MTO. In parallel, it was shown that PLGA-siCD47-MTO markedly increased the intra-tumor infiltration of macrophages and T lymphocytes, both involved in cancer cell clearance. This further proves the expected mechanism of action of PLGA-siCD47-MTO. A gene expression analysis also revealed that variations in gene transcription occurred for the immune response, phagocytosis, cytokine-cytokine receptor interaction, regulation of antigen processing and presentation, the inflammatory response and the T cell receptor signaling pathway. Thus, the authors convincingly proved that PLGA-siCD47-MTO could efficiently elicit an anticancer immune response. The fact that the developed strategy was also effective for melanoma opens up the possibility that different types of human cancer could benefit from this approach.

Cancer stem cells (CSCs) represent the faction of cells thought to confer the ability to initiate/maintain tumors and to be responsible for tumor recurrence and drug resistance. CD44 is considered to be one of the most common CSC surface markers, responsible for the regulation of cancer cell stemness [104]. Moreover, the expression level of CD44 is known to have prognostic values. Zou et al. [98] explored the effects of CD44 silencing (siCD44) in CSC isolated from the human colorectal cancer cell line HCT116 (HCT116-CSC). Following siCD44 delivery to cultured HCT116-CSC, a remarkable inhibition of cell proliferation with a block in the G1/G0 phase of the cell cycle was detected. In parallel, cell apoptosis was also promoted. Cell migration and invasion were down-regulated as well. For in vivo experiments, HCT116-CSCs stably transfected with shRNA-CD44 and sh-NC (non-coding, control) were subcutaneously injected into mice to generate a xenograft model. The authors showed that in the tumor mass obtained from HCT116-CSCs stably transfected with shRNA-CD44, CD44 expression was significantly reduced and this resulted in an evident down-regulation of tumor growth. This observation was in line with the fact that the proliferative marker Ki67 was significantly reduced. Finally, in animals bearing HCT116-CSCs stably transfected with shRNA-CD44, lung metastasis was greatly suppressed compared to control.

The efflux of therapeutic drugs from the tumor cells is one of the most generally acknowledged mechanisms of drug resistance. Among other enzymes, ATPase copper efflux transporter A (ATP7A) mediates drug efflux from the cancer cell. ATP7A is one of the key regulators of the intracellular level of platinum-based drug such as Oxaliplatin (OXA) [105], commonly used to treat colon cancer patients. Based on this knowledge, Zhou et al. [99] delivered to the colon cancer cells HCT116 and LOVO a siRNA (siATP7A) by means of a lipid based delivery system. Besides reaching good down-regulation in the expression of the target gene, the authors could also show that cells treated by siATP7A displayed increased sensibility to OXA. Moreover, the combination of siATP7A and OXA effectively inhibited cell growth and improved apoptosis. Additionally, a down-regulation in cell migration as evaluated by trans-well assays was observed. For the in vivo tests, a delivery material based on the copolymer PEG-PLGA linked to a cationic lipid to encapsulate siATP7A (PEG-PLGA-siATP7A) was chosen. In a subcutaneous xenograft mouse model of colon cancer (HCT116), a good cancer cell uptake with parallel down-regulation in the expression of ATP7 was demonstrated. This resulted in reduced tumor growth and an evident sensitivity to OXA in PEG-PLGA-siATP7A treated animals. Finally, and importantly, no evident adverse effects to major organs of mice were observed. These data suggest the potential feasibility of a siRNA-based approach to increase drug sensitivity to colon cancer cells.

## 4. Clinical Trials

So far, the number of clinical trials involving siRNA for GI tumors are very limited. Additionally, often the patients included are affected by different GI tumors. Thus, we thought it was more logical to group them in a separate paragraph at the end of the present review. The work reported in this section has been summarized in Table 7.

Atu027 is a chemically stabilized siRNA delivered via liposomal particles that targets the expression of protein kinase N3 (PKN3) in the vascular endothelium. PKN3 is a protein kinase C-related molecule thought to be an effector mediating malignant cell growth downstream of activated phosphoinositide 3-kinase (PI3K). The chronic activation of PI3K signal transduction pathway contributes to metastatic cell growth. A phase 1 study of Atu027 was tested in patients with advanced solid tumors, including colon cancer [106,107]. The study demonstrated that Atu027 was well tolerated in the patients. Based on the mode of action, future studies that examine efficiency of combination with traditional cytotoxic drugs are recommended.

CALAA-01 is a cyclodextrin-based polymeric nanoparticle loaded with a siRNA that targets the mRNA of the M2 subunit of ribonucleotide reductase (R2). CALAA-01 is the first targeted, polymer-based nanoparticle carrying siRNA to be systemically administered to humans. A phase Ia [108,109] study reported that CALAA-01 administered to patients with histologically or cytologically confirmed solid malignancy that is metastatic or unresectable, refractory to standard therapy, can provide the targeted delivery of siRNA and is well tolerated.

In the phase 1 clinical study identified with number NCT02227459 [110], the purpose was to evaluate the safety and tolerability of multiple doses of Vitamin A-coupled Lipid Nanoparticle containing siRNA against HSP47 in subjects with moderate to extensive liver fibrosis. HSP47 (Heat shock protein 47) is localized in the endoplasmic reticulum and is a procollagen-specific molecular chaperone required for the biosynthesis and assembly of collagen. So far, no data have been presented.

TKM-080301 is a lipid nanoparticle (LNP) formulation containing siRNA against the PLK1 (polo-like kinase-1) gene product. More specifically, TKM-080301 is a type of LNP formulation, referred to as SNALP (Stable Nucleic Acid Lipid Particles). PLK1 has been validated as a molecular target and a prognostic factor in a variety of cancers. Inhibition of PLK1 activity in proliferating cancer cells rapidly induces mitotic arrest and apoptosis. TKM-080301 was administered to patients with unresectable colorectal, pancreatic, gastric, breast, ovarian and esophageal cancers with hepatic metastases; additionally, patients with primary liver cancer which relapsed after first line treatment were also included. This study (NCT01437007) is upcoming, and no results have been published.

The investigational agent siG12D LODER (Local Drug EluteR) is a miniature biodegradable polymeric matrix that encompasses the anti KRAS^G12D^ siRNA (siG12D) drug, designed to release the drug regionally within a pancreatic tumor, at a prolonged rate of 12-16 weeks. As most pancreatic ductal adenocarcinomas involve mutations in the KRAS oncogene (the most common is G12D), the administration of KRAS^G12D^ siRNA has the potential to be of therapeutic value. In phase I/IIa clinical trials (NCT01188785) [111], patients with locally advanced pancreatic cancer were considered for the intra-tumor implantation of siG12D LODER. Additionally, Gemcitabine was given on a weekly basis, following the siG12D-LODERTM insertion. Ten out of twelve patients demonstrated stable disease and two showed partial response. The combination of siG12D-LODER^TM^ and Gemcitabine was well tolerated, safe and demonstrated a potential efficacy. A Phase 2 Study of KRAS-LODER in combination with chemotherapy in patients with locally advanced pancreatic cancer (NCT01676259), is ongoing.

The purpose of the Atu027 Plus Gemcitabine trials is to evaluate a novel treatment strategy for advanced pancreatic cancer disease by combining Atu027 with the standard chemotherapeutic gemcitabine. The objectives of this clinical trial (NCT018086389) were to evaluate safety and activity of two Atu027 schedules in combination with standard gemcitabine treatment in patients with advanced or metastatic pancreatic adenocarcinoma. The results indicate that Atu027 plus gemcitabine is safe and well tolerated [112]. Moreover, in subjects with metastatic pancreatic cancer, Atu027 is associated with significantly improved outcomes.

## 5. Conclusions

In this review, we summarized some scientific research describing the potential of siRNA-based drugs for GI tumors. So far, the major challenge for siRNA use concerns appropriate delivery to the target cells/tissues. In this regard, some aspects have to be considered. The first deals with the ability of the delivery system to protect siRNA and to deliver it with optimal timing. This task does not seem to be dramatically difficult, judging from the works so far published in oncology regarding GI tract tumors. The second, and more complex task, deals with the ability to reach a specific targeting of tumor cells, leaving the normal cells untouched as much as possible. This aspect is particularly relevant when the systemic delivery of siRNA is necessary, such as in many cases of GI tumors. Only for localized tumors may it be possible to think of an in-situ administration. Obviously, the possibility to detect tumors in the initial phase depends on an effective screening campaign which, for GI tumors such as those of colorectal cancer, is nowadays a reality. The specificity of targeting could be achieved via two approaches: one based on the targeting of antigens exclusively/predominantly expressed on GI tumor cells; the other using siRNAs directed against GI cancer related oncogenes. The identification of optimal molecules to be targeted is, however, not easy since tumor cells may change the surface antigens over time. Additionally, cancer cells frequently share with the normal counterpart similar antigens. Also, the strategy to direct siRNA against cancer related oncogenes may be difficult, as often they are also expressed in normal cells, although to a lesser extent. Regardless of the above difficulties, we think that an ideal level of specificity may be achieved by combining the targeting of specific surface cancer antigens with the use of siRNAs directed against genes predominantly overexpressed in cancer cells, but not in the normal counterpart. In conclusion, despite the as yet unresolved issues in siRNA delivery, we are confident that by considering the above aspects, together with the encouraging works described here, siRNAs have the potential to become novel molecules for GI cancer treatment in the future.

## Figures and Tables

**Table 1:** siRNA delivery systems.

Material	Advantages	Disadvantages
Liposomes	Cheap synthesis Easy functionalization Biodegradable	Tendency to accumulate in the liver and lung if not functionalized Possible induction of mild inflammation
GalNac *	Targeting properties Biodegradable	
Exosomes	Excellent biodistribution and biocompatibility	Production by patient cells
PLGA *	Biocompatible biodegradable FDA approved	Not easy siRNA encapsulation due to PLGA hydrophobic nature
Chitosan	Easy functionalization	Low transfection efficiency and low solubility
Hyaluronic acid	Targeting ability toward cluster determinant 44, over-expressed in cancer cells Biodegradable Biocompatible	Functionalization required to bind siRNA due to its negative electric charge
Dendrimers	Great loading capacity related to the size	Possible toxicity related to particle size
Mesoporous silica	Biocompatible High surface area, the large pore volume, chemical and thermal stability	Functionalization required for targeting purposes
Gold nanoparticles	High surface area to volume ratio, possible multi-functionalization, easy synthesis, non-toxic non-immunogenic	Functionalization required to bind siRNA due to the negative electric charge

* GalNac: N-Acetylgalactosamine; PLGA: Copolymer of polylactic acid (PLA) and polyglycolic acid (PGA).

**Table 2:** siRNA employment in stomach cancer.

Target mRNA	Delivery System	Tumor Model	Ref.
BRCAA1 *	bacteriophage phi29 packaging motor with Folic acid targeting moiety	MGC803 cell line	Cui et al. [48]
AEG-1 *	Lipofectamine	SGC7901 cell line	Jian-bo et al. [49]
Bcl-2 *	Lipofectamine	BGC-823 cell line	Liu et al. [50]
PRL-3 *	Lipofectamine	SGC-7901 cell line	Cao et al. [51]

* BRCAA1: breast cancer-associated antigen 1; AEG-1 astrocyte elevated gene 1; Bcl-2: B lymphocyte/leukemia-2; PRL-3 phosphatase of regenerating liver-3 (PRL-3).

**Table 3:** siRNA employment in pancreatic cancer.

Target mRNA	Delivery System	Tumor Model	Ref.
NGF *	Gold nanoparticles	Panc-1 cell line; mouse subcutaneous model of pancreatic cancer; orthotopic patient-derived xenograft mouse model	Lei et al. [54]
KRAS^G12D^ *	Exosome	Panc-1 cell line; orthotopic mouse model	Kamerkar et al. [55]
PD-L1 *	PLGA	orthotopic model humanized mouse model	Jung et al. [56]
PLK1 *	Polymer	KPC8060 cell line; orthotopic syngenic mouse model (KPC8060); xenograft mouse model (S2-‘13)	Tang et al. [57]
FARSA *	Lipofectamine and porous silicon nanoparticles	SW1990 and Panc-1 cell lines; orthotopic patient-derived xenograft mouse model	Yuan et al. [58]

* NGF: nerve growth factors; KRAS^G12D^: Kirsten rat sarcoma virus mutation G12D; Bcl-2: PD-L1: Programmed death-ligand 1; PLGA: copolymer of polylactic acid and polyglycolic acid; PLK1: Polo-like kinase 1; FARSA: Phenylalanyl-TRNA Synthetase Subunit Alpha.

**Table 4:** siRNA employment in liver fibrosis.

Target mRNA	Delivery System	Liver Fibrosis Model	Ref.
TGF-β1 *	Liposome	CCl_4_-induced murine model of liver fibrosis	Kim et al. [66]
TGF-β1 *	Lipofectamine	HSC-T6	Cheng et al. [67]
PDGFR *	siRNA expression from glial fibrillary acidic protein promoter	CCl_4_-induced murine model of liver fibrosis and bile duct ligation induced chronic rat liver injury	Chen et al. [68]
PDGF α receptor *	Commercial liposome	LX2	Lim et al. [69]
Type I collagen	Liposome	LX2; CCl_4_-induced murine model of liver fibrosis; spontaneous model of mouse biliary fibrosis	Calvente et al. [70]
Type I collagen	Lipid bound to vitamin A	LI-90; CCl_4_-induced murine model of liver fibrosis	Toriyabe et al. [71]
MMP2 *	Lipid bound to vitamin A	HSC-T6	Li et al. [72]
TIMPs *	Electroporation	HSC isolated from normal livers of Sprague–Dawley rats.	Fowel et al. [73]
STAT3 *	Exosomes	HSC isolated from healthy mouse; CCl_4_-induced murine model of liver fibrosis	Tang et al. [74]

* TGF-β1: Transforming growth factor TGF-β1; PDGFR: platelet-derived growth factor receptor; MMP2: matrix metalloproteinase 2; TIMPs: Tissue inhibitors of metalloproteinases; STAT3: signal transducer and activator of transcription 3.

**Table 5:** siRNA employment in hepatocellular carcinoma.

Target mRNA	Delivery System	Tumor Model	Ref.
FMRP *	Carbon dots conjugated with the aptamer AS1411	HepG2	Zhao et al. [75]
Survivin and VEGF *	Galactose-modified trimethyl chitosan-cysteine	Xenograft mouse model of HCC	Han et al. [76]
eEF1A1, eEF1A2 *, E2F1	PDPG polymer bound to galactose	HuH7; Xenograft mouse model of HCC	Perrone et al. [77]
Pin1	GalNac-siRNA embedded into a gel	Orthotopic mouse model	Zhao et al. [78]
RRM2 *	liposome-polycation-DNA complexes linked to anti-EGFR * Fab’	Orthotopic mouse model	Gao et al. [79]
TERT *	PEGylated liposomes conjugated with antibodies against TfR * and HIR *	Xenograft mouse model of HCC	Hu et al. [80]
VEGF *	Polymer containing urocanic acid-modified galactosylated trimethyl chitosan	QGY-7703; mouse xenograft subcutaneous model	Han et al. [81]
Survivin	PEG/PEI conjugated with RGD *	Subcutaneous mouse model	Wu et al. [82]

* FMRP: Fragile X mental retardation protein; VEGF: Vascular Endothelial Growth Factor; eEF1A1/2: eukaryotic elongation factor 1A1/2; RRM2: ribonucleotide reductase M2; EGFR: Epidermal growth factor receptor; TERT: telomerase reverse transcriptase; TfR: transferrin receptor; HIR: human insulin receptor; RGD: tripeptide arginine glycine aspartic acid.

**Table 6:** siRNA employment in colorectal cancer.

Target mRNA	Delivery System	Tumor Model	Ref.
CD73 *	Chitosan linked to TAT *-Hyaluronic acid	CT26; subcutaneous xenograft mice model	Khesth et al. [94]
CPTA1 *	Exosome linked to iRGD *	HCT116; xenograft subcutaneous mouse model	Lin et al. [95]
PD-1 *	Attenuated Salmonella	Mouse xenograft subcutaneous model	Lu et al. [96]
CD47 *	PLGA *	Xenograft mouse model	Zhang et al. [97]
CD44 *	Liposome	HCT116-CSC; xenograft subcutaneous mouse model	Zou et al. [98]
ATP7A *	PEG-PLGA linked to a cationic lipid	HCT116 and LOVO; subcutaneous xenograft mouse model	Zhou et al. [99]

* TAT-HA: HIV cell-penetrating peptide;.CD73: enzyme able to convert AMP to adenosine; CPTA1: Carnitine palmitoyltransferase 1A; iRGD: peptide that favors extravasation and interaction with integrins; PD-1: immunosuppressive receptor able to down-regulate T cell activation; PLGA: copolymer of polylactic acid and polyglycolic acid; CD47: cluster determinant 47; CD44: cluster determinant 44; ATP7A: ATPase copper efflux transporter A.

**Table 7:** siRNA in clinical trials.

Target mRNA/Delivery System	Clinical Trial/Results	Disease	Number
PKN3 */liposome	Phase 1/well tolerated	Colorectal cancer	NCT00938574
RRM2 */polymer	Phase 1a/well tolerated	Unresectable solid tumors	NCT00689065
HSP47 */lipid	Phase I	Liver fibrosis	NCT02227459
PLK1 */lipid	Phase I	Unresectable colorectal, pancreas, gastric, breast, ovarian and esophageal cancers with hepatic metastases	NCT01437007
KRAS^G12D^ */	Phase I/IIa Phase II	Pancreatic carcinoma	NCT01188785 NCT01676259
PKN3 */polymer	Phase Ib/IIa	metastatic pancreatic adenocarcinoma	NCT018086389

* PKN3: expression of protein kinase N3; HSP47: Heat shock protein 47; PLK1: polo-like kinase-1; KRAS^G12D^: Kirsten rat sarcoma virus mutation G12D.

## Data Availability

Data sharing not applicable.
